# Untargeted metabolomics analysis reveals *Mycobacterium tuberculosis* strain H37Rv specifically induces tryptophan metabolism in human macrophages

**DOI:** 10.1186/s12866-022-02659-y

**Published:** 2022-10-17

**Authors:** Guohui Xiao, Su Zhang, Like Zhang, Shuyan Liu, Guobao Li, Min Ou, Xuan Zeng, Zhaoqin Wang, Guoliang Zhang, Shuihua Lu

**Affiliations:** 1 National Clinical Research Center for Infectious Diseases, Guangdong Provincial Clinical Research Center for Tuberculosis, Shenzhen Third People’s Hospital, Southern University of Science and Technology, Shenzhen, 518112 China; 2 School of Basic Medical Sciences, Guangdong Medical University, Dongguan, China

**Keywords:** *mycobacterium tuberculosis*, Metabolome, THP-1, Tryptophan metabolism, Virulence

## Abstract

**Background:**

Tuberculosis (TB) caused by *Mycobacterium tuberculosis* (*M. tb*) remains a global health issue. The characterized virulent *M. tb* H37Rv, avirulent *M. tb* H37Ra and BCG strains are widely used as reference strains to investigate the mechanism of TB pathogenicity. Here, we attempted to determine metabolomic signatures associated with the Mycobacterial virulence in human macrophages through comparison of metabolite profile in THP-1-derived macrophages following exposure to the *M. tb* H37Rv, *M. tb* H37Ra and BCG strains.

**Results:**

Our findings revealed remarkably changed metabolites in infected macrophages compared to uninfected macrophages. H37Rv infection specifically induced 247 differentially changed metabolites compared to H37Ra or BCG infection. Kyoto Encyclopedia of Genes and Genomes (KEGG) pathway analysis revealed H37Rv specifically induces tryptophan metabolism. Moreover, quantitative PCR (qPCR) results showed that indoleamine 2,3-dioxygenase 1 (IDO1) and tryptophan 2,3-dioxygenase 2 (TDO2) which converts the tryptophan to a series of biologically second metabolites were up-regulated in H37Rv-infected macrophages compared to H37Ra- or BCG-infected macrophages, confirming the result of enhanced tryptophan metabolism induced by H37Rv infection. These findings indicated that targeting tryptophan (Trp) metabolism may be a potential therapeutic strategy for pulmonary TB.

**Conclusions:**

We identified a number of differentially changed metabolites that specifically induced in H37Rv infected macrophages. These signatures may be associated with the Mycobacterial virulence in human macrophages. The present findings provide a better understanding of the host response associated with the virulence of the *Mtb* strain.

**Supplementary Information:**

The online version contains supplementary material available at 10.1186/s12866-022-02659-y.

## Background

Tuberculosis, mainly caused by *Mycobacterium tuberculosis,* is the most common infectious disease worldwide, leading to an estimated 1.8 million deaths in 2020 worldwide. *M. tuberculosis* is one of the most successful pathogens for overcoming the host defense system and adapting to long-term residence in the host. *M. tuberculosis* can initially reside and replicate in human alveolar macrophages [1]. Macrophage apoptosis is a common defense strategy for hosts against intracellular mycobacteria [2–4]. Human alveolar macrophage apoptosis induced by bacilli is associated with the virulence of the bacteria. The virulent *M. tb* strain H37Rv was found to induce less human alveolar macrophage apoptosis than avirulent or attenuated bacilli (*M. tb* H37Ra and BCG) [5, 6]. The virulent strain H37Rv grew more rapidly in human alveolar macrophages than attenuated strains [6, 7]. Animal model infection also demonstrated that the virulent strain H37Rv exhibits greater replication in vivo than the aviruent strain H37Rv, which resulted in a higher bacterial burden of *M. tuberculosis* in the lungs and other organs [8–10].

Obtaining primary human alveolar macrophages from healthy individuals is challenging since bronchoscopy is an invasive collection method. It is difficult to obtain enough cells for experiments from one individual. Primary alveolar macrophages are always heterogeneous and highly variable among donors. These factors have led to poor repeatability of experiments and made them difficult to interpret. Human macrophage-like THP-1 cells are an alternative to mimic the interaction of *M. tuberculosis* with primary macrophages [11, 12]. It has been demonstrated that phorbol-myristate acetate (PMA)-treated THP-1 cells display similar behaviors to primary human alveolar macrophages in terms of bacterial uptake, bacterial intracellular survival and replication [13]. A previous study reported that the avirulent strains H37Ra and *M. bovis* BCG induce more apoptosis in THP-1 cells than the virulent strain H37Rv, consistent with the behavior of alveolar macrophages [14].

*M. tb* H37Rv and H37Ra have been widely used as models of virulent and avirulent strains, respectively, in laboratory studies. BCG remains the only available vaccine for tuberculosis and is also used as an attenuated reference strain in labs. Although these three strains displayed similar morphology under a microscope and a very close genetic evolutionary relationship, infection of H37Rv causes disease in animals, whereas the H37Ra and BCG strains do not. It is important to identify mycobacterial virulence determinants to aid our understanding of the pathogenesis of TB. Metabolic reprogramming in immune cells plays an important role in the regulation of the immune response to infectious pathogens [15]. There are a number of literatures reporting the differences in transcriptome level of macrophages infected by different *M. tb* strains []. However, to date, there are no studies investigating differences of metabolism in macrophages in response to different *M.tb* strains. Here, to determine the H37Rv-specifically induced metabolic responses which may associated with the Mycobacterial virulence in human macrophages, we compared the metabolome in THP-1-derived macrophages following exposure to the *M. tb* H37Rv, *M. tb* H37Ra and BCG strains.

## Results

### Overview of identified metabolites among all group samples

**Fig. 1:**
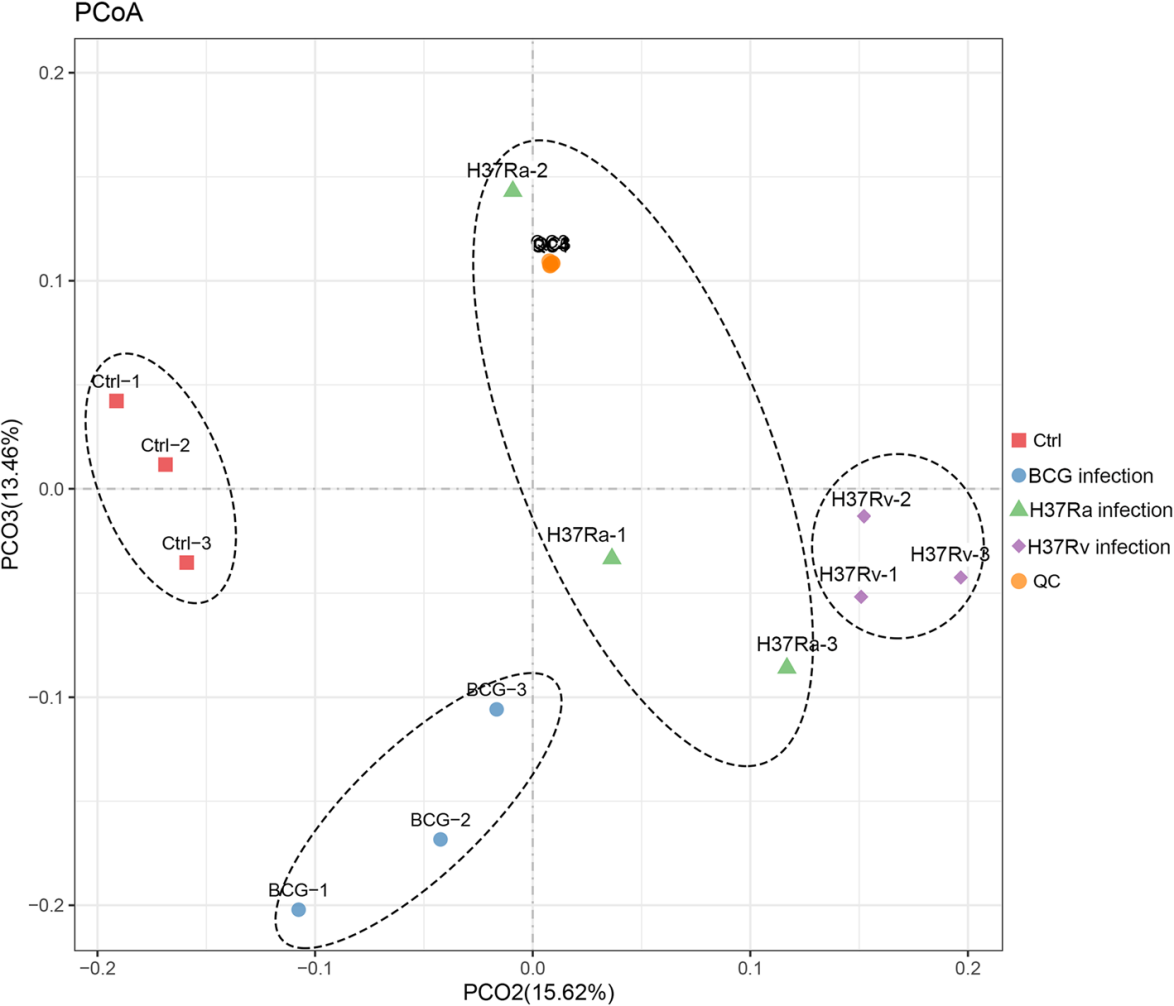
Principal co-ordinates analysis (PCoA) of samples. QC represents quality control

Macrophages undergo profound metabolic reprogramming in response to microbial infection, which is critical for defining the fate of Macrophages function in the context of mycobacterial survival [15]. Here, we characterized the profile of metabolites of THP-1 derived macrophages in response to the strains of BCG, H37Ra and H37Rv using LC-MS/MS analysis. To improve metabolite coverage, we used the positive ion (POS) and negative ion (NEG) modes in mass spectrometry detection. A total of 10,935 and 6641 peaks were identified by the POS and NEG modes, respectively (Table 1 and Table S2). Among them, a total of 2677 metabolites were annotated (Table 1 and Table S2). For a preliminary visualization of differences among different groups of samples, PCoA was calculated with Bray-Curtis dissimilarity. The result of PCoA that showed that control, BCG-infected, H37Ra-infected and H37Ra-infected groups of sample were clearly separated in the plot (Fig. 1), indicating that there were differences in metabolites among these groups.

**Table 1:** Overview of all identified metabolites in this study

Model	Peaks	MS1 Spectra	MS2 Spectra	Annotations(MS1 + MS2)
POS	10,935	282	1145	1727
NEG	6641	70	880	950

### THP-1-derived macrophages displayed significantly changed profile of metabolites in response to different *M. tb* strains infection

**Fig. 2:**
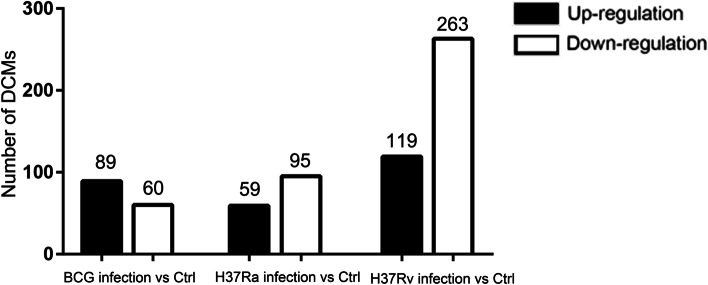
Number of DCMs in BCG-, H37Ra- and H37Rv-infected THP-1 derived macrophages compared to uninfected macrophages

To determine how the metabolic profiling of macrophages change in response to different strains infection, we performed comparisons of the metabolome between infected macrophages and uninfected controls. Differentially changed metabolites (DCM) were screened using a combination of the *p*-value of Student’s *t*-tests with the VIP score. The metabolites with |Log2 (fold change)| ≥ 1, *p*-value< 0.05 and VIP ≥ 1 were considered different. As shown in Fig. 2, a total of 382 (with 119 upregulated and 263 downregulated), 154 (with 59 upregulated and 95 downregulated), and 149 (with 89 upregulated and 60 downregulated) differentially changed metabolites were identified in H37Rv-infected, H37Ra-infected and BCG-infected macrophages compared to uninfected macrophages. The details of these identified DCMs were provided in Supplementary table S3-S5. The THP-1 derived macrophages displayed more DCMs in response to virulent strain H37Rv than response to avirulent H37Ra and attenuated BCG. These differential metabolites may provide new clues for understanding the role of intracellular metabolites in the complex regulation of macrophages function.

### Identification of H37Rv specifically induced DCMs in THP-1-derived macrophages

**Fig. 3:**
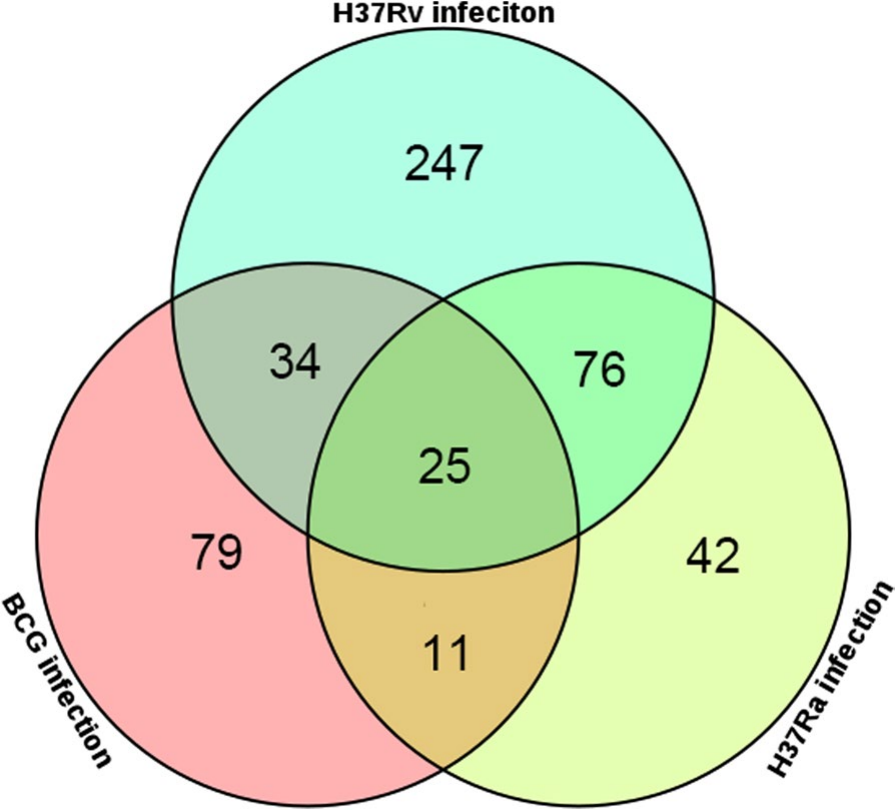
Venn diagram displaying the numbers of common and specific DCMs in the comparisons of BCG infection vs. the control, H37Ra infection vs. the control and H37Rv infection vs. the control

**Fig. 4:**
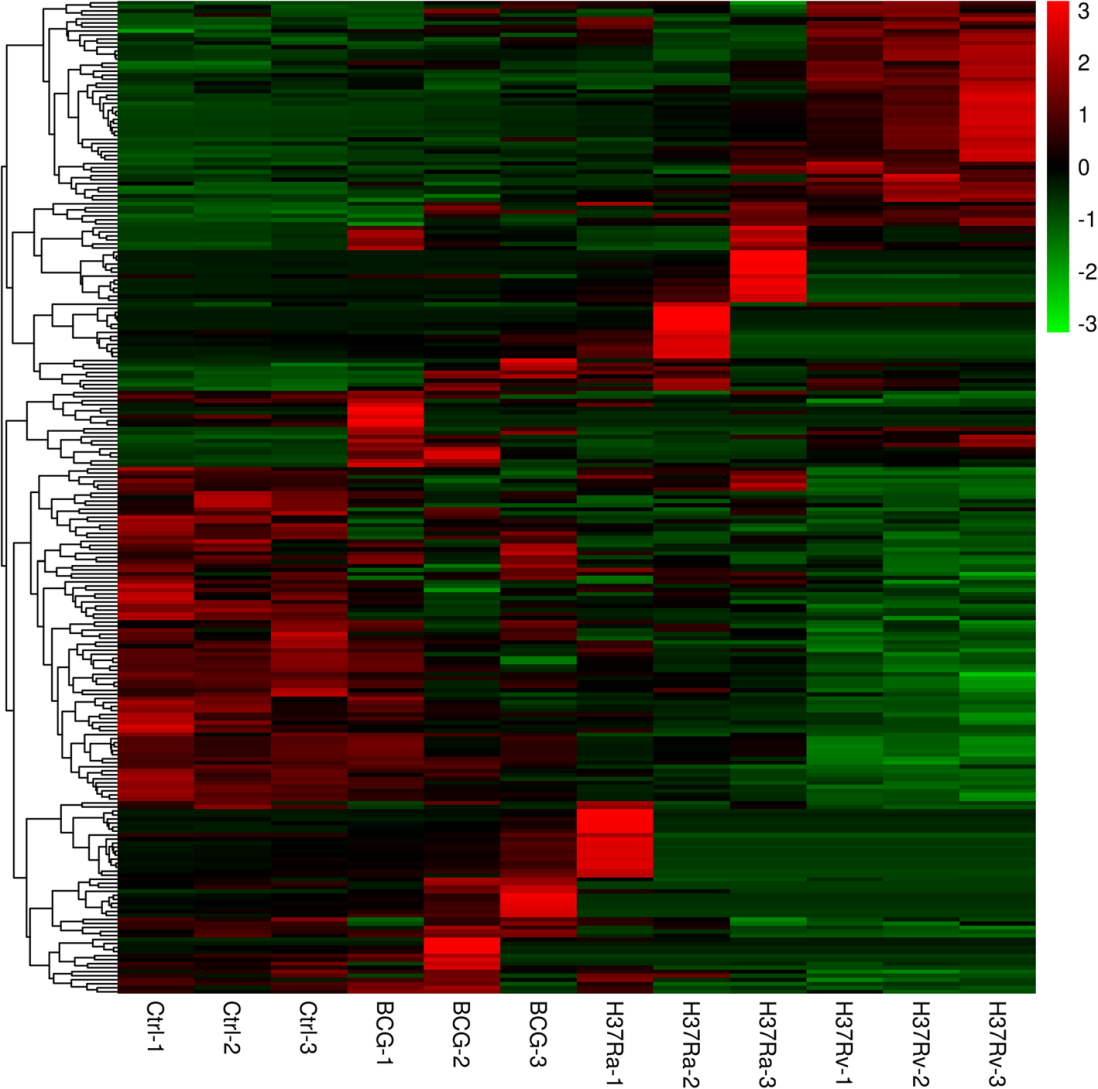
The heatmap of H37Rv specifically induced DCMs. Red represents high expression and green corresponds to low expression

Although virulent strain H37Rv share closed genome with H37Ra and BCG, only infection of H37Rv causes disease in animals. We though that H37Rv may be able to induce specifically DCMs in macrophages during infection. To determine the specific metabolites induced by H37Rv infection, we compared the DCMs in H37Rv-infected macrophages with H37Ra- and BCG-infected macrophages. A Venn diagram was constructed with DCMs in macrophages infected with different strains (Fig. 3). The result revealed that 25 DCMs shared across all groups. There are 247, 42, and 79 DCMs specifically presenting in H37Rv-, H37Ra- and BCG-infected macrophages, respectively. As a comparison, H37Rv infection induced much more DCMs than H37Ra or BCG infection. The heatmap showed the expression of H37Rv-specific DCMs (Fig. 4). Details of all 247 DCMs were listed in Supplementary Table S6 in order of expression level. Among the 247 H37Rv-specific DCMs, 84 were downregulated and 163 were upregulated. Of the 247 H37Rv-specific DCMs, 39 metabolites had annotations. All 39 annotated metabolites were listed in Table 2 in order of expression level. Among them, 8 DCMs were up-regulated and 31 DCMs were down-regulated (Table 2).

**Table 2:** H37Rv specific DCMs in macrophages

Annotation	log2_FC	*p*-value
Quinolinic acid	3.444867916	0.012258307
(R)-2-O-Sulfolactate	3.085257487	0.000222446
L-Formylkynurenine	2.346917917	0.000472705
L-Kynurenine	1.625380001	0.000528383
4-Methylumbelliferyl acetate	1.557321092	0.022054592
2-Hydroxy-3-oxoadipate	1.39306116	0.002737469
2-Aminophenol	1.230185203	0.021124038
12-Keto-tetrahydro-leukotriene B4	1.043843279	0.009570237
Deltaline	−1.008242179	0.002507065
Carmustine	−1.009502891	0.007213718
Corticosterone	−1.067454697	0.006600774
Pyrophosphate	−1.142726141	0.003398876
Himbacine	−1.385102859	0.027800095
Cytidine	−1.507338991	0.011402052
Linoleate	−1.716931395	0.007682331
4-Oxoglutaramate	−1.775861036	0.023332493
Succinic acid	−1.81138885	0.018232547
NAD	−2.093087731	0.000856109
O-Phosphoethanolamine	−2.10681556	0.043010552
Methabenzthiazuron	−2.295552471	0.001788476
L-Lactic acid	−2.551250819	0.002712846
Rifampicin	−2.610450994	0.00019059
L-Proline	−3.401789	0.017554737
Nicotinate D-ribonucleoside	−3.538851201	0.004664017
2,3-Bisphosphoglycerate	−21.22035171	0.000816274
Penicillin V	−21.28311057	8.55E-06
CMP	−21.29007334	0.003457214
Malate	−21.45167026	0.000344413
4-Methylumbelliferone	−21.5202691	0.029428915
IDP	−21.62423728	0.016617187
Colchicine	−21.68545183	0.000765791
Nordiazepam	−21.77968227	1.09E-06
3,5-Dibromo-L-tyrosine	−22.05515368	2.26E-05
Hydrocinnamic acid	−22.05629256	4.83E-06
3-Aminoisobutanoic acid	−22.5273831	0.000171486
N-Acetyl-L-aspartic acid	− 23.76527459	0.000794018
Dihydrobiopterin	−24.92189005	0.001019856
Norethindrone acetate	−24.96437137	0.000112173
Guanidinosuccinic acid	−26.05630378	2.90E-07

### KEGG pathway analysis of H37Rv specifically induced DCM in THP-1 derived macrophages

**Fig. 5:**
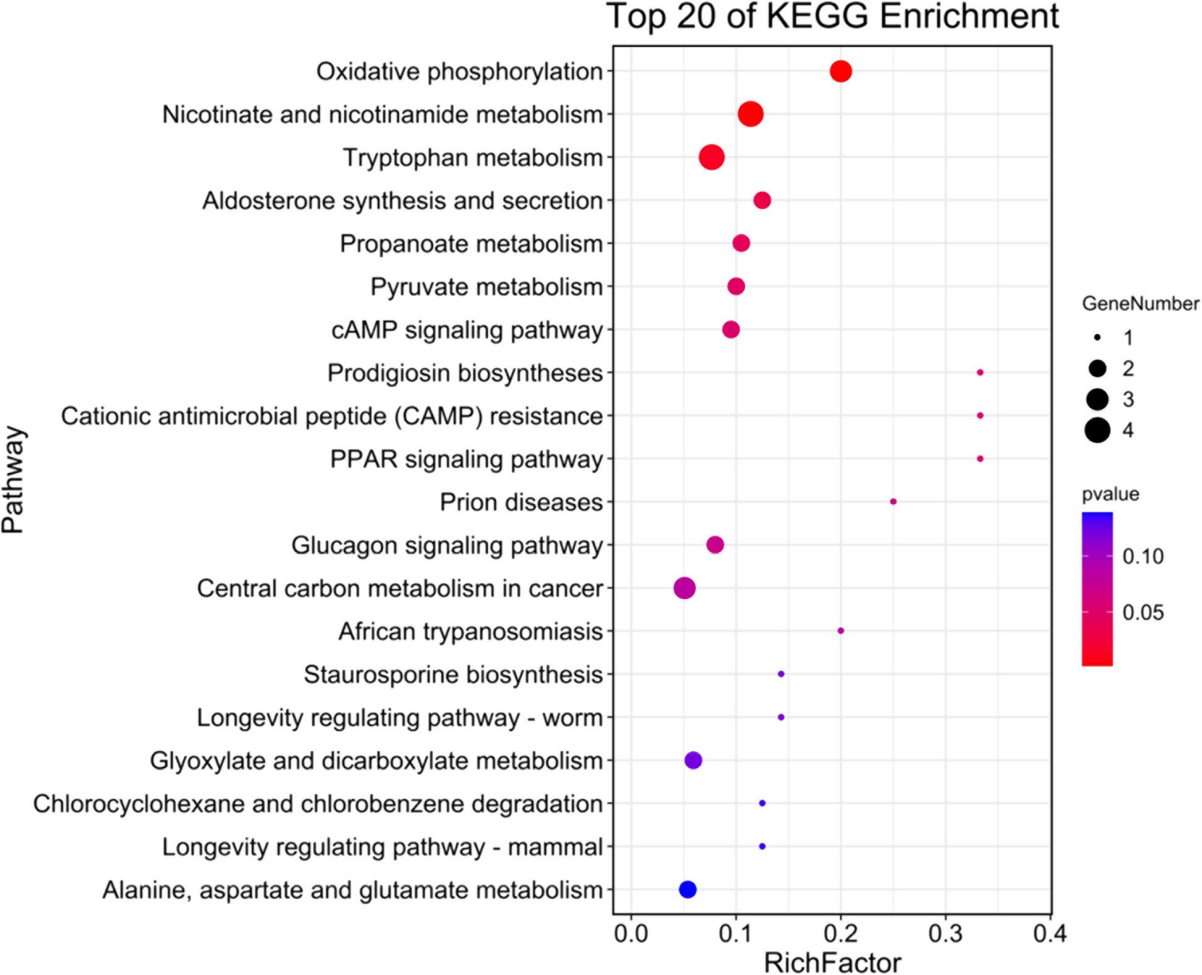
KEGG pathways analysis of H37Rv-specific DCMs in macrophages [20]

These DCMs specifically induced by H37Rv infection may contribute to mycobacterial survival. To explore the H37Rv-specific DCMs involved in possible pathways, we conducted KEGG pathway analysis with the 39 annotated metabolites. As shown in Fig. 5 and Supplementary Table S7, These DCMs were significantly enriched in six metabolic pathways, including oxidative phosphorylation (OXPHOS), nicotinate and nicotinamide metabolism, Trp metabolism, aldosterone synthesis and secretion, propanoate metabolism and pyruvate metabolism. OXPHOS, nicotinate and nicotinamide metabolism and trp metabolism were the three most significantly enriched pathways. Two (NAD, succinic acid and pyrophosphate), four (NAD, succinic acid, quinolinic acid (QUIN) and nicotinate D-ribonucleoside) and four metabolites (QUIN, L-Formylkynurenine (LFKYN), L-Kynurenine (KYN) and 2-Aminophenol) involved in above top three metabolism pathways, respectively. The detail expression of these metabolites were showed in Table 2. The NAD, succinic acid and pyrophosphate were down-regulated in H37Rv infected macrophages, suggesting that H37Rv infection may reduce OXPHOS. While the DCMs involved in tryptophan metabolism were all up-regulated, suggesting that H37Rv infection specifically accelerates trp metabolism.

### Determination of expression of genes involved in trp metabolism by qPCR

**Fig. 6:**
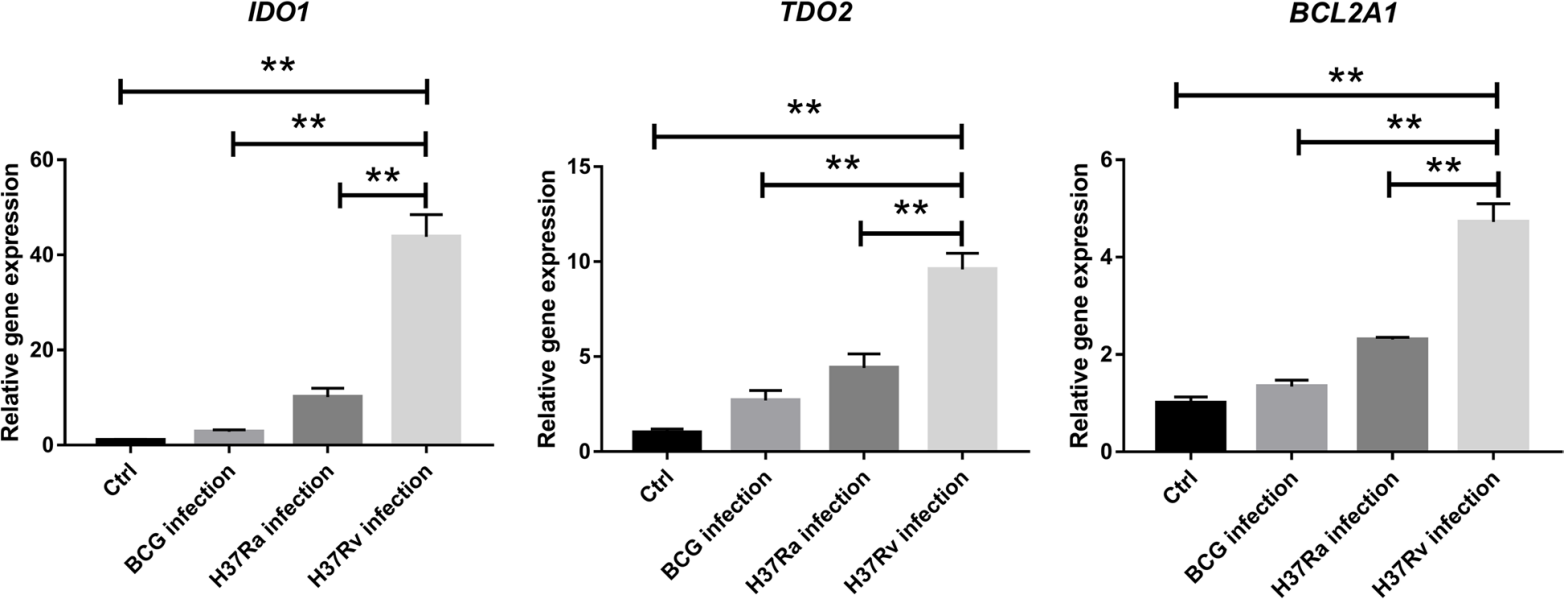
Determination of expression of genes involved in trp metabolism. mRNA level of target genes was detected by qPCR. Vertical bars represent the means ± S.D. (*n* = 3). * *p*<0.05, ** *p*<0.01

Trp is an essential amino acid which cannot be synthesized by humans. Trp metabolism plays an important role in immune regulation [21]. We thus further determined whether mRNA expression of genes related to trp metabolism were consistent with up-regulated DCMs. The first step of Trp metabolism is the conversion of Trp to N-formyl-L-kynurenine, by indoleamine 2,3-dioxygenase (IDO) or tryptophan 2,3-dioxygenase 2 (TDO2) [22]. The qPCR results showed that both *IDO1* and *TDO2* are up-regulated in H37Rv-infected macrophages compared to H37Ra-infected, BCG-infected or uninfected macrophages (Fig. 6), consisting with the significantly increased LFKYN in H37Rv-infected macrophages. A recent study reported that IDO suppresses apoptosis by repressing BCL2A1 expression in oral squamous cell carcinoma [23]. We observed significantly increased expression of *BCL2A1* in mRNA level inH37Rv-infected macrophages compared to H37Ra-infeced, BCG-infected or uninfected macrophages (Fig. 5). Similarly, these results indicate that H37Rv infection may inhibit apoptosis of macrophages by up-regulation of *BCL2A1*.

## Discussion

Macrophage activation plays a critical role in host defense against *M. tb* infection [24, 25]. However, macrophages frequently fail to eradicate the infection, and mycobacteria employ these cells as a reservoir for replication [26, 27]. The virulent strain H37Rv, the avirulent strain H37Ra and attenuated BCG have been widely used as reference strains in research. Although they share a high degree of genome identity, infection with only H37Rv causes disease in animals. Recent evidence has proven that metabolic remodeling is involved in the regulation of the immune response against *M. tb* infection [28]. However, understanding of the metabolic reprogramming of macrophages in response to different *M. tb* strains infection is very limited. Thus, the aim of the present study was to identify and characterize the H37Rv-specific induced metabolites through the comparison of metabolome in macrophages infected with H37Rv, H37Ra and BCG. Since the virulent strain H37Rv was more capable of surviving in macrophages, we anticipated that infection with H37Rv would induce weaker responses in macrophages than infection with the strains H37Ra or BCG. Instead, we found that H37Rv infection induced more DCM in the THP-1-derived macrophages than H37Ra- or BCG-infected macrophages. The identified 284 DCMs specifically enriched in H37Rv-infected macrophages, suggesting virulent strain modulates host metabolic reprogramming during infection, which may contribute to bacterial replication.

The ability of Mtb to induce and alter cellular death pathways in alveolar macrophages is a critical factor for determination of the outcome of infection [29]. *M. tuberculosis* kills infected macrophages through necroptosis, a programmed cell death that enhances mycobacterial replication and dissemination [4, 29, 30]. The tuberculosis necrotizing toxin (TNT) is a secreted NAD^+^ glycohydrolase that induces necrosis in infected macrophages through NAD^+^ hydrolysis [31, 32]. Our results revealed significantly decreased NAD^+^ in the H37Rv-infected macrophages, suggesting that the virulent strain H37Rv may induce stronger macrophage necroptosis than H37Ra or BCG. We found that all the three strains could encode the TNT protein. We speculate the H37Rv NAD^+^ glycohydrolase exhibits stronger NAD^+^ hydrolyzing activity, resulting in decreased NAD^+^ concentration during infection. Our results indicate that replenishment of NAD^+^ may inhibit necroptosis of macrophages caused by Mtb infection.

Tryptophan is an essential amino acid in human protein synthesis. It is also required for microbial growth. Therefore, IDO is thought to be antibacterial by depleting available tryptophan [33]. However, *M. tuberculosis* can synthesize its own tryptophan de novo, which may be an adaptation for its survival during the degradation of tryptophan by IDO in host phagocytes [34]. Thus, IDO production has little effect on mycobacterial metabolism, but may affect the host’s protective immune response. As effector cells in the immune system, macrophages play important roles in both innate and adaptive immunity [24]. According to the polarization state of macrophages, it is generally accepted that macrophages can be divided into two main classical phenotypes, pro-inflammatory M1 macrophages and anti-inflammatory M2 macrophages [35]. The pro-inflammatory M1 phenotype tend to killing and elimination of pathogens, while anti-inflammatory M2 phenotype leads to immune tolerance, induction of regulatory T cells, tissue repair and fibrosis [36]. Macrophages favors polarization to an immunosuppressive phenotype (M2) rather than a pro-inflammatory phenotype in the presence of high IDO [37]. In the present study, we observed the virulent strain H37Rv specifically induced a higher Kyn concentration and up-regulation of *IDO1* and *TDO2* inTHP-1 derived macrophages, suggesting that virulent *Mtb* may induce anti-inflammatory M2 phenotype of macrophages through depletion of Trp by IDO1 and TDO2 in host cells, which is benefit for bacterial survival.

It was reported that induction of host IDO inhibits T-cell functions and generates regulatory T cells through depletion of Trp, leading to immune suppression or tolerance [38].Targeting Trp catabolism in combination with additional therapies improve efficacy of cancer immunotherapy [39]. Previous studies revealed that pulmonary TB patients had significantly higher expression of IDO1 and Kyn concentration in serum compared to healthy individuals [40–42]. Gautam et al. demonstrated that suppression of IDO activity resulted in reduced bacterial load and increased lymphoid follicles and proliferation of pulmonary lymphocytes, which contributed to increased host survival [43]. We thus thought that inhibition of IDO1 may be an effective and clinically relevant host-directed therapy for TB.

Macrophage apoptosis plays an important role in successful immunity to intracellular pathogens [44]. However, *M. tb* has evolved various mechanisms to inhibit macrophage apoptosis for its replication and dissemination [4, 29, 45]. Recently, Qiaoping Zheng et al. reported that inhibition of IDO induces apoptosis of OSCC cells through repressing BCL2A1 expression. Here, we found the anti-apoptotic gene *BCL2A1* significantly increased in H37Rv infected macrophages, suggesting that virulent strain may inhibit apoptosis through specifically enhancing tryptophan catabolism. However, substantial experiments need to be done in the future to confirm this hypothesis.

## Conclusions

This study has shown that virulent strain H37Rv infection specifically induced numbers of metabolites in macrophages. Our findings revealed a tryptophan metabolism pathway specifically induced by virulent Mtb strain in THP-derived macrophages. Although the H37Rv-specific tryptophan metabolism pathway were validated by qPCR results, extensive experiments are lacking to validate our observations. For example, we can measure the variation of tryptophan by knockout or knockdown the genes involved in tryptophan metabolism and determine their impact on Mtb H37Rv survival in macrophages. Taken together, our findings provide new insights into the understanding of host-pathogen interaction.

## Materials and methods

### Cell line, bacterial strains and growth conditions

The THP-1 cells used in this study were purchased from the American Type Culture Collection (ATCC) and grown in RPMI 1640 (Gibco, Carlsbad, CA) supplemented with 10% (V/V) heat-inactivated fetal bovine serum (Gibco, Carlsbad, CA) in a humidified 5% CO_2_ atmosphere at 37 °C. The *M. tuberculosis* strains H37Rv and H37Ra and *M. bovis* bacillus Calmette-Guerin (BCG) were provided by the China CDC and were grown in Middlebrook 7H9 liquid medium containing 10% OADC (oleic acid, albumin, dextrose, and catalase) and 5% glycerol. The bacterial cells were suspended in phosphate-buffered saline (PBS) at a concentration of 1 × 10^8^ CFU/mL and stored at − 80 °C.

### Infection of THP-1 cells and sample collection

THP-1 cells were treated with 100 ng/mL phorbol 12-myristate 13-acetate (PMA) for 24 h for differentiation into macrophages and then washed three times with fresh culture medium. Cells were rested 24 h before infection. For metabolomic analysis, 1 × 10^7^ macrophages were infected with 1 × 10^8^ mycobacteria to achieve a multiplicity of infection (MOI) of 1:10 in 10 cm dishes for 4 h in triplicate. For qPCR analysis, 3 × 10^6^ macrophages were infected with 3 × 10^7^ mycobacteria to achieve a MOI of 1:10 in 6 cm dishes for 4 h in triplicate. Extracellular bacteria were removed by washing with fresh culture medium. The supernatants were removed 24 h post-infection (p.i.), followed by washing twice with PBS. For RNA isolation, the macrophages were harvested with TRIzol. For metabolite extraction, the macrophages were harvested by scraping, then moved to a centrifuge tube at 4 °C 1000 g for 1 min. Discard the supernatant and quenched by liquid nitrogen and then stored at − 80 °C. In parallel, uninfected macrophages with similar treatment were used as controls.

### LC-MS/MS Analysis

Metabolite extraction and measurement were performed by Guangzhou Genedenovo Biotechnology Co., Ltd. (Guangzhou, China). The collected samples were transferred to 2 mL EP tubes and dissolved in 1 mL of extract solvent (acetonitrile/methanol/water = 2:2:1). After the addition of extract solvent, the samples were vortexed for 30 s, homogenized for 2 min at 60 Hz. The operation was repeated twice. The obtained solution were centrifuged at 4 °C for 10 min at 12000 rpm. 850 μL of the supernatant from each sample were transferred to another 2 mL tube and concentrated to dry in vacuum. Samples were dissolved in 300 μL of 2-chlorobenzalanine solution. The supernatants were filtered through 0.22 μm membrane to obtain the prepared samples for LC-MS. The quality control (QC) samples were prepared by mixing 20 μL of the supernatants from each samples.

The LC-MS/MS analyses were performed according to the previous study with minor modifications [46]. Chromatographic separation was accomplished in an Thermo Vanquish system equipped with an ACQUITY UPLC® HSS T3 (150 × 2.1 mm, 1.8 μm, Waters) column maintained at 40 °C. The temperature of the autosampler was set as 8 °C. Gradient elution of analytes was carried out with 0.1% formic acid in water (B2) and 0.1% formic acid in acetonitrile (A2) or 5 mM ammonium formate in water (B1) and acetonitrile (A1) at a flow rate of 0.25 mL min^− 1^. Injection of 2 μL of each sample was done after equilibration. An increasing linear gradient of solvent A (v/v) was used as follows: 0 ~ 1 min, 2% A2/A1; 1 ~ 9 min, 2% ~ 50% A2/A1; 9 ~ 12 min, 50% ~ 98% A2/A1; 12 ~ 13.5 min, 98% A2/A1; 13.5 ~ 14 min, 98% ~ 2% A2/A1; 14 ~ 20 min, 2% A2-positive model (14 ~ 17 min, 2% A1-negative model).

The electro-spray ionization multistage mass spectrometry (ESI-MSn) experiments were executed on the Thermo Q Exactive Focus mass spectrometer with the spray voltage of 3.5 kV and − 2.5 kV in positive and negative modes, respectively. Sheath gas and auxiliary gas were set at 30 and 10 arbitrary units, respectively. The capillary temperature was 325 °C. The analyzer scanned over a mass range of m/z 81-1000 for full scan at a mass resolution of 70,000. Data dependent acquisition (DDA) MS/MS experiments were performed with HCD scan. The normalized collision energy was 30 eV. Dynamic exclusion was implemented to remove some unnecessary MS/MS information.

### Qualitative and quantitative analysis of metabolites

The MS raw data were converted to the mzML format using ProteoWizard (version 3.0.8789) and processed by R package XCMS (version 3.2). After data processing, a data matrix consisting of the retention time, mass-to-charge ratio (m/z) values, and peak intensity was generated. OSI-SMMS software (version 1.0, Dalian Chem Data Solution Information Technology Co. Ltd., Dalian, China) was used for peak annotation after XCMS data processing with in-house MS/MS database.

### Principal co-ordinates analysis (PCoA)

For a preliminary visualization of differences among different groups of samples, PCoA among all sample groups was calculated with Bray-Curtis dissimilarity using R package models (http://www.r-project.org/).

### Differential metabolites analysis

Differentially changed metabolites were screened using a combination of the *p*-value of Student’s *t*-tests with the VIP (variable importance of the projection) score. The metabolites with |Log2 (fold change)| ≥ 1, *p*-value< 0.05 and VIP ≥ 1 were considered different.

### Kyoto encyclopedia of genes and genomes pathway analysis

To determine the significantly enriched pathways, the different metabolites were mapped to KEGG database for pathway enrichment analysis [20]. Calculated *p*-values were corrected for FDR, with FDR ≤0.05, as a threshold. Metabolic pathways satisfying this condition were defined as significantly enriched pathways in the differential metabolites.

### Quantitative PCR

Total RNA was extracted from the collected samples with Total RNA Kit I (OMEGA, R6834-02). The complementary DNA was synthesized using ClonExpress Ultra One Step Cloning Kit (Vazyme, C115-02) according to the manufacturer’s instructions. Quantitative PCR reaction mixture was prepared using SYBR Green HiScript II Q RT SuperMix for qPCR Kit (Vazyme, R223-01). The qPCR reaction was performed on ABI ViiA7 Real-Time thermal cycler (Thermo Fisher, ABI). Primers for qPCR were listed in Supplementary Table 1. The relative expression of gene of interest was calculated using the 2^-ΔΔCT^ method [47]. qPCR analysis was performed with three technical replicates. The data represent the means ± standard errors (*n* = 3). *P* value < 0.05 was considered as significant difference.

## Supplementary Information

**Additional file 1.**

**Additional file 2.**

**Additional file 3.**

**Additional file 4.**

**Additional file 5.**

## Data Availability

All data generated or analyzed during this study have been included in this published article and its supplementary information files. If someone wants to request further information or data, please contact Dr. Guoliang Zhang with e-mial: szdsyy@aliyun.com.

## Abbreviations

TB: Tuberculosis
BCG: Bacillus Calmette-Guerin
DCMs: Differentially changed metabolites
KEGG: Kyoto Encyclopedia of Genes and Genomes
qPCR: Quantitative PCR
IDO1: Indoleamine 2,3-dioxygenase 1
TDO2: Tryptophan 2,3-dioxygenase 2
PAM: Phorbol-myristate acetate
Trp: Tryptophan
POS: Positive
NEG: Negative
OXPHOS: Oxidative phosphorylation
NAD: Nicotinamide adenine dinucleotide
QUIN: Quinolinic acid
LFKYN: L-Formylkynurenine
KYN: L-Kynurenine
PCD: Programmed cell death
OADC: Oleic acid, albumin, dextrose, and catalase
PBS: Phosphate-buffered saline
MOI: Multiplicity of infection
VIP: Variable importance of the projection
QC: Quality control
ESI-MSn: Electro-spray ionization multistage mass spectrometry
DDA: Data dependent acquisition
PCoA: Principal co-ordinates analysis
FDR: False discovery rate
