# Supplementary material for: Untargeted metabolomics analysis reveals Mycobacterium tuberculosis strain H37Rv specifically induces tryptophan metabolism in human macrophages
Source: BMC Microbiol. 2022 Oct 17;22:249. doi: 10.1186/s12866-022-02659-y (PMC9575276; doi:10.1186/s12866-022-02659-y)
Supplement: Supplementary file 1 — Additional file 1. [file 12866_2022_2659_MOESM1_ESM.docx]

**Supplementary materials**

**Untargeted metabolomics analysis reveals
Mycobacterium tuberculosis strain H37Rv specifically induces tryptophan metabolism in human macrophages**

Guohui Xiao^1#^, Su Zhang^1#^, Like Zhang^2#^, Shuyan Liu^1#^,Guobao Li^1^, Min Ou^1^, Xuan Zeng^1^ , Zhaoqin Wang^1*^, Guoliang Zhang^1, 2*^, Shuihua Lu^1*^

1. National Clinical Research Center for Infectious Diseases, Guangdong Provincial Clinical Research Center for Tuberculosis, Shenzhen Third People's Hospital, Southern University of Science and Technology, Shenzhen, 518112, China

2. School of Basic Medical Sciences, Guangdong Medical University, Dongguan, China

# These authors contributed equally to this work.

*Correspondence author.

Dr. Zhaoqin Wang, [wangzhaoqin6666@163.com](mailto:wangzhaoqin6666@163.com)

Dr. Guoliang Zhang, [szdsyy@aliyun.com](mailto:szdsyy@aliyun.com)

Dr. Shuihua Lu, [lushuihua66@126.com](mailto:lushuihua66@126.com)

**Table S1:** All primers used in this study

**Table S1 Primers used in the study**

| Name | Sequence (5’-3’) | For purpose |
| --- | --- | --- |
| β-actinF | CATGTACGTTGCTATCCAGGC | Reference genes amplification |
| β-actinR | CTCCTTAATGTCACGCACGAT | Reference genes amplification |
| H-TDO2F | CGGTGGTTCCTCAGGCTATC | Amplification of *TDO2* |
| H-TDO2R | CTTCGGTATCCAGTGTCGGG | Amplification of *TDO2* |
| BCL2A1 | TTACAGGCTGGCTCAGGACT | Amplification of *BCL2A1* |
| BCL2A1 | AGCACTCTGGACGTTTTGCT- | Amplification of *BCL2A1* |
| H-IDO1 F | AGCAGCGTCTTTCAGTGCTT | Amplification of *IDO1* |
| H-IDO1 R | AGAGCTTTCACACAGGCGTC | Amplification of *IDO1* |
